# Apical bud manipulation and integrated nutrient management enhance yield and profitability of cabbage (*Brassica oleracea var. capitata* L.) in Northwestern Ethiopia

**DOI:** 10.1038/s41598-026-41149-3

**Published:** 2026-02-24

**Authors:** Yohannes Gelaye

**Affiliations:** College of Agriculture and Natural Resources, Debre Markos University, P.O. Box 269, Amhara, Ethiopia

**Keywords:** Cabbage phenology, Head size distribution, Organic manure fertilizer, Ecology, Ecology, Environmental sciences, Plant sciences

## Abstract

Cabbage (*Brassica oleracea var. capitata* L.) is an important vegetable crop in Ethiopia, yet its productivity remains low due to poor soil fertility and suboptimal agronomic practices. This study assessed how integrating nutrient management with apical bud manipulation influences cabbage growth, yield, and profitability in northwestern Ethiopia. A field experiment was conducted during the 2021/2022 season at two locations in East Gojjam Zone, Ethiopia, using a randomized complete block design in a 3 × 4 factorial arrangement with three bud levels (one, two, and three) and four farmyard manure (FYM) rates (0, 2.5, 5, and 7.5 t ha^− 1^). Growth, yield, and quality traits were recorded and statistically analyzed. Significant interactions occurred between bud retention and FYM rates for all traits. The two-bud + 5 t ha^− 1^ FYM treatment produced the highest marketable yield (41.8 t ha^− 1^), total yield (43.1 t ha^− 1^), and medium head size (31.8 t ha^− 1^). Total yields at 2.5 and 7.5 t ha^− 1^ FYM were consistently lower across bud levels, with two buds yielding moderately at both rates, whereas one- and three-bud treatments resulted in further reductions reinforcing the superiority of the two-bud + 5 t ha^− 1^ FYM combination. This treatment also generated the highest net return (1917.59 USD ha^− 1^). Integrating moderate organic nutrient inputs with proper bud management improves cabbage yield and profitability. The application of 5 t ha⁻¹ FYM with two-bud retention is recommended for sustainable cabbage production in northwestern Ethiopia and comparable agro-ecologies.

**Supplementary Information:**

The online version contains supplementary material available at 10.1038/s41598-026-41149-3.

## Introduction

Cabbage (*Brassica oleracea* var. *capitata* L.) belongs to the family Brassicaceae and is a biennial crop characterized by superimposed leaves forming a compact head^1^. It is widely cultivated for its edible head, which provides vitamins, minerals, and bioactive compounds beneficial to human health^2^. *Brassicaceae* vegetables constitute an important and diverse group of globally cultivated crops, including broccoli, Brussels sprouts, cauliflower, kale, mustard greens, and collard greens^3^.

As a cool-season crop, cabbage is grown worldwide during both spring and fall, and is consumed in fresh and processed forms^4^. Its wide adaptability to varied climatic and soil conditions contributes to its global importance, and Ethiopia provides particularly suitable environments for high-quality production^5^. Optimal performance occurs around 18 °C, whereas temperatures exceeding 27 °C may lead to head cracking and poor formation^6^. The crop grows best in light sandy soils mixed with clay, rich in organic matter, and with a pH of 5.5–6.5^7^. Soil pH below 4.5 or above 6.5 can impair leaf quality, and saline soils increase susceptibility to blackleg disease^8^. Cabbage also contains antioxidant, anti-inflammatory, and antibacterial properties, which support its use in traditional medicine. Nutritionally, it is high in water content and dietary fiber^9^.

Cabbage is cultivated in more than 90 countries, with China, India, and South Korea serving as the world’s leading producers^10^. In Africa, major producers include Kenya, Egypt, Ethiopia, Niger, and South Africa, where the crop plays a key role in supporting smallholder farmers’ livelihoods and national food security^11^. In Ethiopia, cabbage ranks second among vegetable crops after chili in area cultivated and total production^12^. According to the 2017/18 CSA report, cabbage production in Ethiopia, the Amhara region, and eastern Gojjam is 38,681.45 t, 6,276.43 t, and 1,988.69 t, respectively, with average yields of 6.25, 7.0, and 9.05 t ha⁻¹, compared with the global average of 10.40 t ha^− 1^^13^. These yield gaps suggest that productivity remains below potential and that improved cultivation practices and input use are needed.

Owing to the high cost and limited availability of chemical fertilizers, most smallholder farmers in tropical regions rely on suboptimal fertilizer application and poor farming practices^14^. This has led to soil nutrient depletion, reduced crop yields, and declining soil fertility. In Ethiopia, fertilizer application rates remain below recommended levels and do not meet crop nutrient demands^15^. This situation indicates the need for integrated soil fertility management approaches that combine organic and inorganic nutrient sources to improve crop performance^16^. Organic fertilizers such as garden manure remain essential, especially for resource-constrained farmers.

Soil properties vary widely across regions, affecting cabbage quality and necessitating the selection of suitable production environments. In the Sinan and Debre Markos districts, the Copenhagen Market variety is commonly grown in backyard plots without the use of technologies such as fertilization or bud number management. Although such practices remain basic, farmers are increasingly interested in practical innovations to enhance productivity. Although research is limited, studies show that cabbage stems produce multiple buds, and secondary buds can form additional edible heads after the primary head is harvested^17^. Harnessing these secondary buds could increase total yield without expanding cultivated land, yet this practice remains poorly documented and understudied.

Organic fertilizers release nutrients slowly and have relatively low nutrient concentrations^18^. Thus, integrating farmyard manure (FYM) application with bud number management is expected to enhance productivity and improve the cost-efficiency of cabbage cultivation. The study area, characterized by integrated crop–livestock systems, provides a reliable supply of FYM that reduces dependence on costly inorganic fertilizers. FYM releases nutrients gradually, improving soil structure, root development, and long-term soil fertility^19^. However, FYM alone is insufficient to achieve maximum cabbage yields^20^. Combining FYM with bud number management provides a practical, locally feasible approach to enhance cabbage yield and support sustainable production.

Despite these opportunities, vegetable production in Ethiopia remains suboptimal due to limited access to improved varieties, inadequate agronomic practices, and constraints from biotic and abiotic stresses^21^. Although improved horticultural varieties have been developed, adoption remains low. Consequently, the lack of improved agronomic practices including nutrient management and bud regulation continues to constrain cabbage production.

To address these gaps, this study was conducted to evaluate the combined effects of FYM application rates and apical bud manipulation on cabbage yield and quality. The specific objectives were to:

- Determine the optimal bud number for achieving high and marketable cabbage yields.
- Assess the effects of different FYM rates on cabbage growth, yield, and quality.
- Identify the most economically viable combination of bud number management and FYM rate for sustainable cabbage production in northwestern Ethiopia.

## Materials and methods

### Description of the study area

**Fig. 1:**
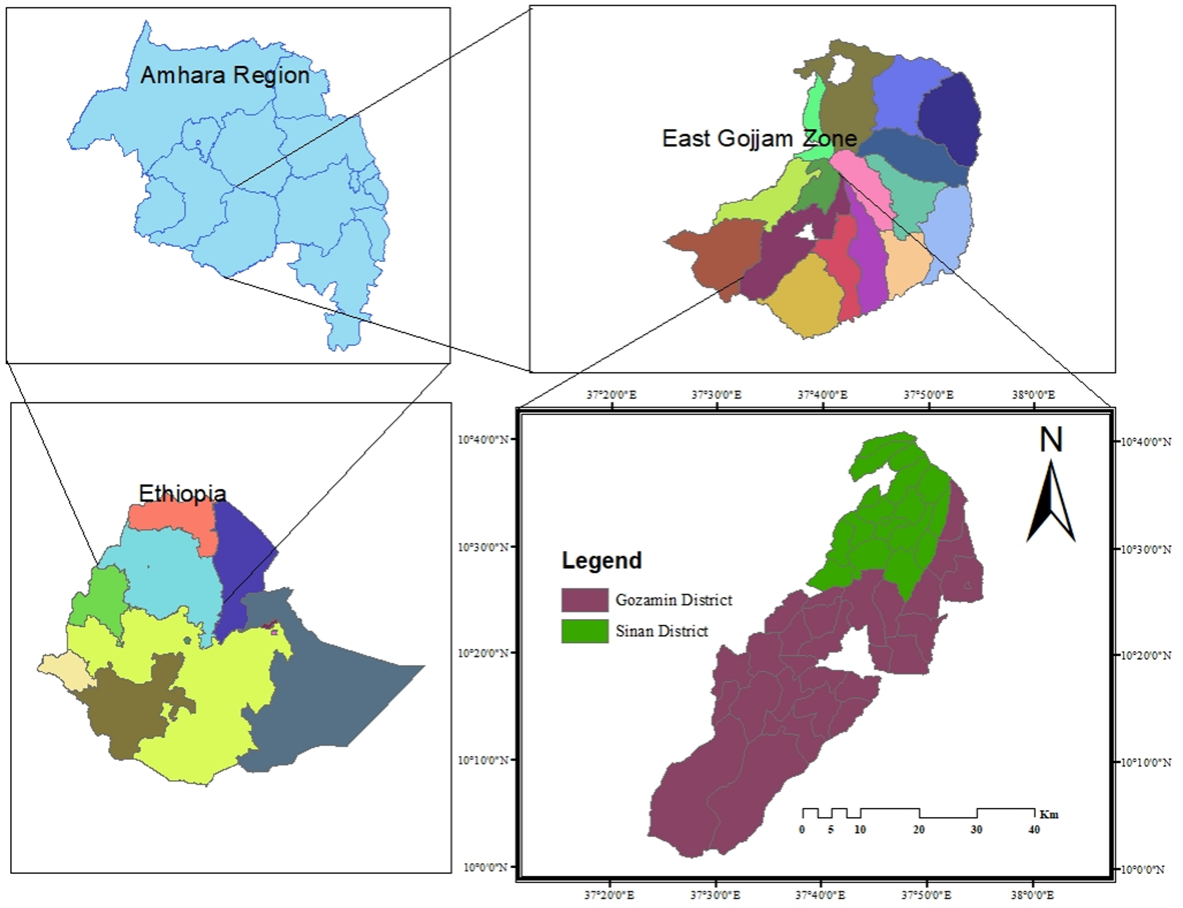
Map of the study locations.

Under rain-fed conditions, the experiment was carried out in two locations in East Gojjam: Sinan (L1), Gedamawit Kebele on a farmer’s field, and Debre Markos (L2), Debre Markos University Research and demonstration site (Gozamin), in 2021/2022 (Fig. 1). Gedamawit Kebele (Sinan) is geographically located between latitudes of 10.7118° or 10° 42’ 42” north and elevations of 37.8435° or 37° 50’ 37” east, and its elevation varies in the range of 2350–3358 meters above sea level. Debre Markos is also located between 10° 19’ 60.00” N Longitude: 38° 00’ 0.00” E and the altitude vary from 2350 to 2500 m above sea level. Even though both locations have a variety of soil types, nitisol is the most prominent^22^ (Table 1).

All the locations have relatively similar annual rainfalls of 1380 mm and minimum and maximum temperatures of 15 °C and 22 °C, respectively (considering months not included in the table) (Table 2). Furthermore, the rainy season in both locations lasts from mid-May to mid-September, with the most rain falling in July and August.

**Table 1:** Soil characteristics of experimental sites. Source: Debre Markos University Choke Watershed project, 2021/2022.

Locations	Constituents	Amount present	Category
L_1_	Soil texture	14: 64: 22 (Sand, Clay, Silt %)	Clay
	pH	4.8	Strongly acidic
	CEC (Cation exchange capacity)	20.61 cmol kg^− 1^	Medium
L_2_	Soil texture	12: 56: 20	Clay
	pH	5.5	Acidic
	CEC	23.54 cmol	Medium

**Table 2:** Mean air temperature, monthly rainfall, soil temperature, relative humidity, and soil moisture in the Sinan and Debre Markos areas. Source: Debre Markos University Choke Watershed project, 2021/2022 (updated).

Experimental sites	Cropping season months	Mean monthly rainfall (mm)	Mean air temperature (°C)		Soil temperature/°C/	RH (%)	Soil moisture (m^3^/m^3^)
			Min	Max.			
L_1_	June	165	14.30	15.5	18.10	89.02	0.28
	July	397	14.35	15.29	17.32	86.14	0.25
	August	143	14.29	15.45	17.97	87.02	0.27
	September	145	14.77	16.05	18.40	80.04	0.51
	October	45	14.53	15.75	18.19	75.29	0.36
L_2_	June	*	14.27	15.4	*	91.4	*
	July	*	14.30	15.27	*	88.7	*
	August	*	14.23	15.01	*	87.3	*
	September	*	14.09	16.02	*	80.1	*
	October	*	14.31	15.70	*	77.8	*

Note (*): The missing data for L2 resulted from the unavailability of measurements during the experimental period.

### Experimental materials, treatments, and design

#### Experimental materials

The Copenhagen market variety was planted in the study, and farmyard manure was used as a fertilizer source.

Copenhagen market cabbage seeds are a Danish heirloom that was introduced in 1909 by H. Hartman and Co^23^. This early variety produces round, and solid heads that measure 15–20 cm (6–8”) in diameter, making it the largest early round-head cabbage for summer harvest.

#### Description of farmyard manure preparation

**Fig. 2:**
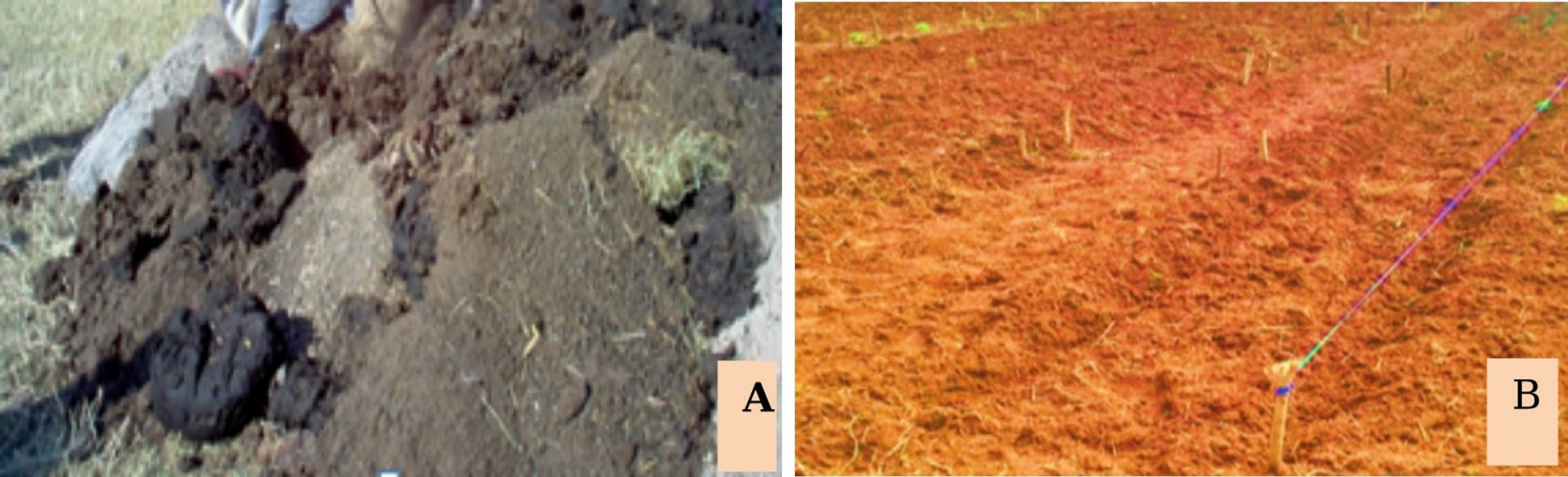
FYM (A) and field (B) preparations.

FYM, specifically cow dung, was sourced from the livestock production unit of Debre Markos University. The collected material was stored for a period of two months in a shaded pit to enhance composting efficiency, minimize nutrient loss, and facilitate preliminary site preparation activities (Fig. 2). Prior to transplanting cabbage seedlings, the required quantity of well-decomposed FYM measured according to the experimental treatment levels was uniformly applied at both the Debre Markos and Gedamawit field sites, two months in advance of planting.

Throughout the composting and application processes, standard scientific protocols for manure management were meticulously followed to ensure the preservation of its agronomic quality. These procedures aimed to enhance compost quality by increasing organic matter, improving cation exchange capacity and soil friability, improving water retention and infiltration, and reducing the risk of soil-borne pests and pathogens.

FYM improves soil structure and is used in farming as a natural fertilizer, and it improves the soil’s ability to retain more water and nutrients. It also boosts soil microbial activity, which improves mineral supply and plant nutrition.

Compared to chemical fertilizers, FYM has been reported to be weak, as its decomposition process produces harmful gases that pollute the atmosphere^24^. Additionally, the availability of certain micronutrients is reduced, and when compared to fertilizer, FYM necessitates a higher cost per unit weight of nutrients during handling, storage, and application^25^.

#### Experimental treatment and design

The experimental treatments consisted of three levels of bud management: no pinching (control), pinching to retain two buds, and pinching to retain three buds. These were combined with four FYM) application rates: 0, 2.5, 5.0, and 7.5 tons per hectare, resulting in a total of twelve treatment combinations. The study was arranged in a RCBD with three replications, following a 3 × 4 factorial treatment structure. FYM rates of 2.5, 5.0, and 7.5 t ha^–1^ were chosen as they align with ranges commonly used by smallholder farmers and recommended in local guidelines. While no single national standard exists, 5–10 t ha^–1^ of well-decomposed FYM is generally advised to enhance soil structure and nutrient supply. Hence, these selected rates cover both lower and upper practical limits, allowing evaluation of incremental FYM effects on cabbage growth and yield under field conditions.

Each experimental plot had a net area of 2.16 m^2^, with intra-row and inter-row plant spacing set at 0.3 m and 0.4 m, respectively. Buffer distances of 0.5 m between adjacent plots and 1.0 m between blocks were maintained to minimize treatment interference and ensure uniform management. The twelve treatment combinations were randomly assigned to the plots within each block, resulting in a total of 36 experimental plots (12 treatments × 3 replications).

### Experimental procedures and management activities

**Fig. 3:**
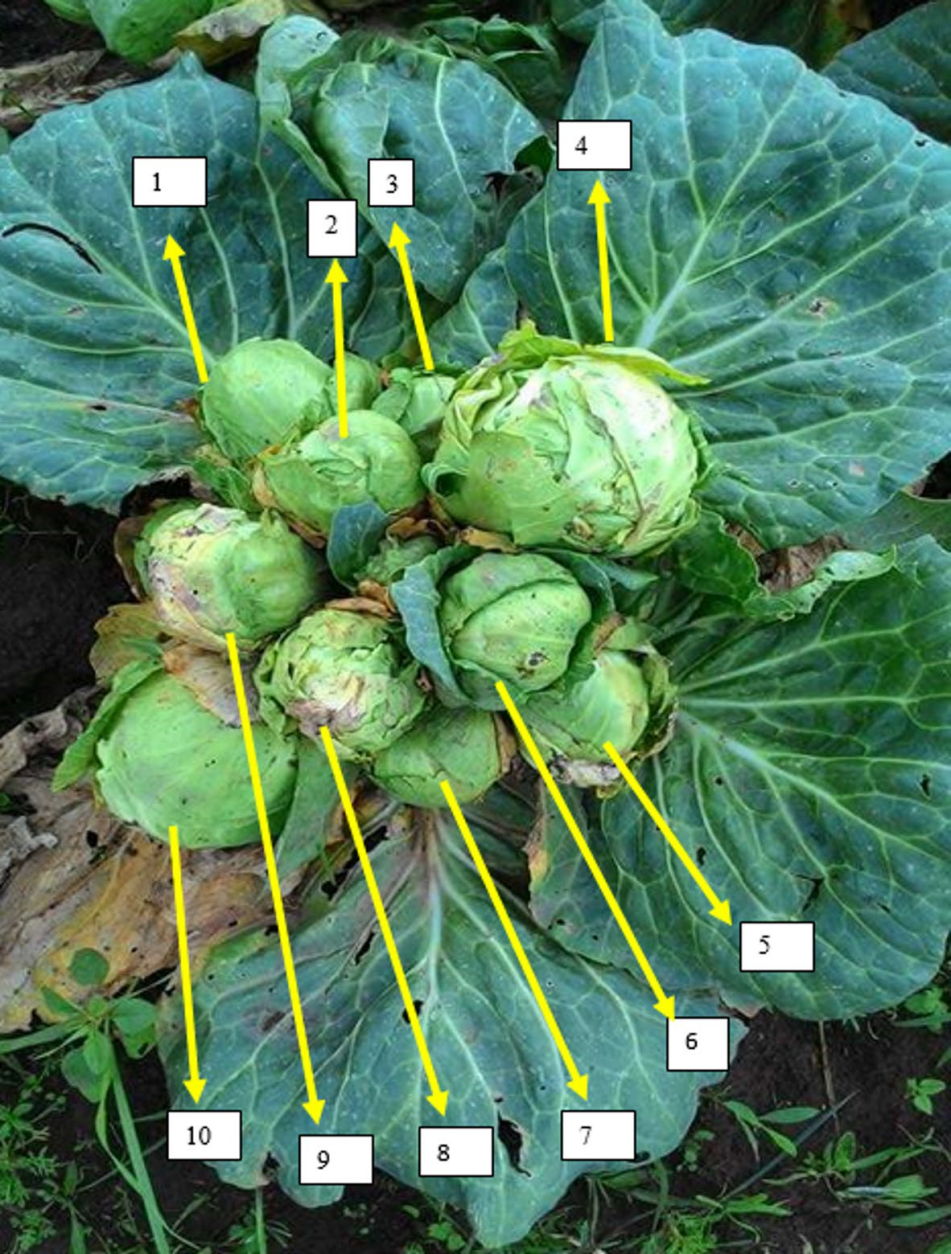
Pinched cabbage crop.

The nursery bed was made with finely prepared soil and well-decomposed FYM^1^, and the size of the bed was 1.0 × 3.0 m. According to the recommendation, cabbage seeds were sown at the Debre Markos nursery site, and seed sowing was done right in line^26^. Cabbage seedlings were considered ready for transplanting when they attained a height of approximately 15 cm and developed 3 to 4 true leaves, typically within 4 to 6 weeks after sowing. Transplanting was conducted on well-prepared raised beds designed to ensure adequate drainage and optimal root development. Each main plot covered a gross area of 2.0 m × 2.4 m, totaling 4.8 m². The number of rows and plants per plot was determined based on the specific treatment combinations assigned. Bud manipulation was conducted in a two-step process to optimize cabbage growth. Initial pinching of apical buds was performed at the time of transplanting to control early vegetative growth, followed by additional bud management 45 days after transplanting to further regulate source–sink balance. For pinched treatments, apical sections of seedlings were removed by cutting or manually pinching 2 to 3 cm from the shoot tip to regulate bud number, while control seedlings remained unpinched. This practice allowed for consistent establishment of the designated bud treatments across all experimental plots^27^. Thus, following successful seedling establishment, newly initiated buds were thinned, with two to three buds per plant depending on the treatment (Fig. 3). The pinching or cutting point was close to the leaf nodes and was performed with care to avoid damaging the small buds. FYM was prepared by collecting animal manure and composting it in a pit for a month^28^. One month before planting, composted FYM was applied to the soil and incorporated into the soil following the combination and arrangement of treatments^29^. Urea fertilizer was applied evenly at the recommended dose of 100 kg/ha in all treatments^30^. Other management practices, such as replanting and refilling, weed control, and insect pest and disease control, were applied equally to all plots based on the cropping package’s general recommendations.

### Data collection

The respective yield and yield-related parameter data were collected from the middle row, with plants in the border row left aside to avoid border effects.

1. Phenological parameters.

Days to 50% head initiation (HI) (number): Recorded when half of the plants in a net plot were heads.

Days to 90% maturity of heads (HM) (number): It was recorded from the date of transplantation until 90% of the heads of the net plot reached maturity.

2. Growth parameters.

Plant Height (PH) (cm): At approximately 90% physiological maturity of the cabbage heads, plant height was measured by placing a ruler vertically from the soil surface to the apex of the longest outer leaf of each individual plant. For each plot, six representative plants were randomly selected, and their heights were recorded and averaged. The mean plant height per plot, expressed in centimeters (cm), was then used for subsequent statistical analysis.

3. Yield parameters.

Head Weight (HW) (g): The heads were weighed with a sensitive balance, the fresh weight of each plant’s head was recorded and expressed in g, and the mean values were used for further analysis.

MY (t ha^− 1^): Marketable yield (MY) was determined by harvesting cabbage heads from the net plot area that were free from insect infestation, disease symptoms, and mechanical damage, and that exceeded a minimum individual weight of 0.5 kg. These heads were weighed using a precision digital balance, and the mean weight per plot was calculated. The resulting values were subsequently converted to tons per hectare (t ha^− 1^) for statistical analysis.

Unmarketable yield (UY), expressed in tons per hectare (t ha^− 1^), was quantified by collecting cabbage heads that were either diseased, mechanically damaged, physically injured, or weighed less than 0.5 kg. These non-marketable heads were weighed using a precision balance, and the total weight per plot was recorded. The resulting values were then converted to a per-hectare basis for further statistical analysis.

Total yield (TY), expressed in tons per hectare (t ha^− 1^), was computed by summing the weights of both marketable and unmarketable cabbage heads harvested from each plot. The combined yield values were then converted to a per-hectare basis for statistical analysis.

4. Quality parameters.

**Fig. 4:**
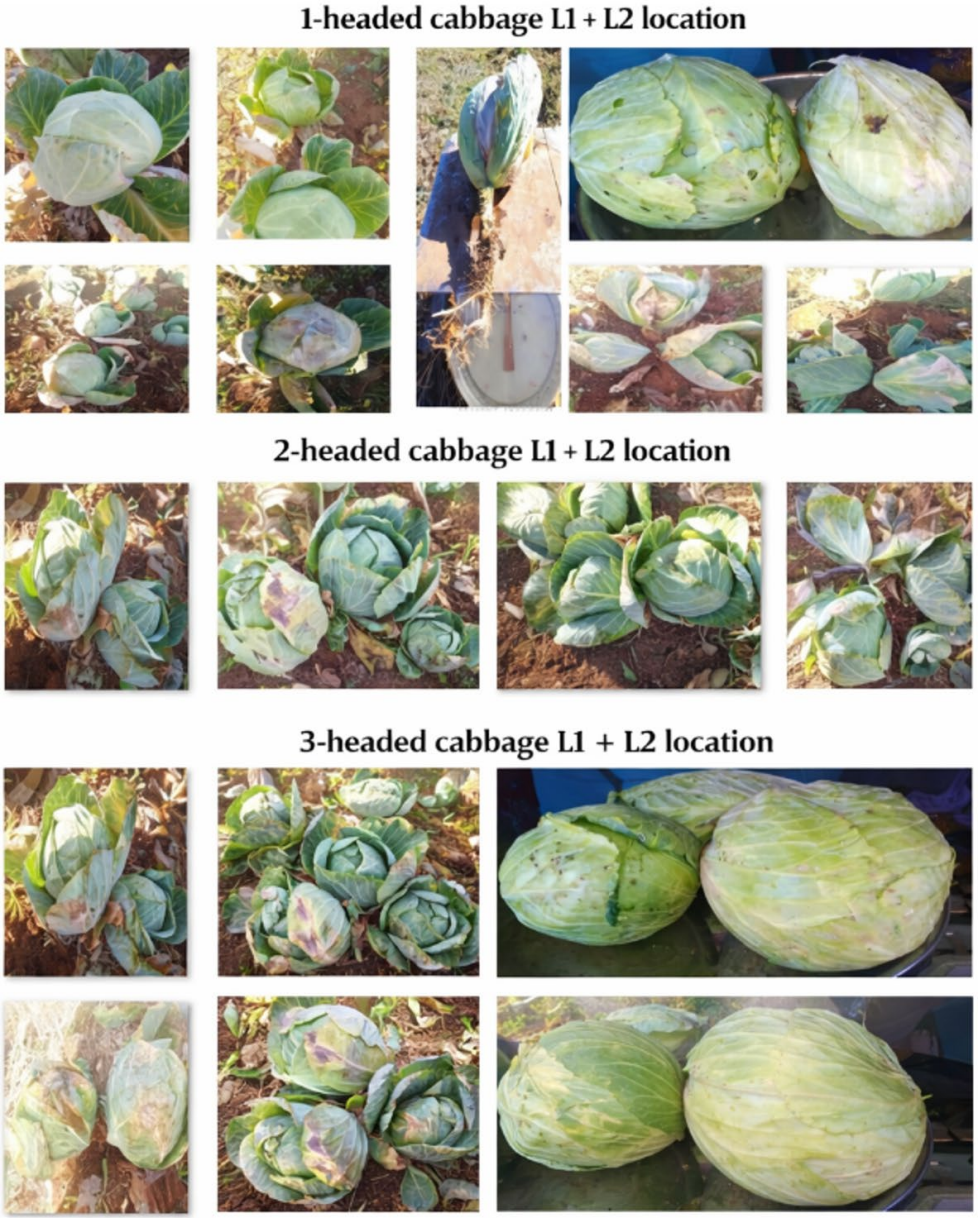
Photos taken during experimentation in two location (1-headed, 2-headed, and 3-headed cabbages.)

Compactness index (Ctn) (%) was determined following standardized measurement protocols. For head size distribution by weight (t ha^− 1^), cabbage heads were categorized based on their individual mass into three classes: large (> 1.0 kg), medium (0.5–1.0 kg), and small (< 0.5 kg), in accordance with the classification system. The heads harvested from the net plot area were weighed using a precision balance (Fig. 4), and the total weight for each size category was calculated and expressed on a per-hectare basis.

### Data analysis

Data analysis was performed using the Statistical Analysis System (SAS) software version 9.2. Initially, separate analyses were conducted for each experimental site to account for location-specific variation. Homogeneity of error variances was assessed using a variance ratio test based on the error mean squares for all evaluated traits; all ratios were below the threshold value of three, confirming sufficient homogeneity across sites and justifying the aggregation of data. Accordingly, a comprehensive combined analysis was performed. Prior to conducting analysis of variance (ANOVA), the assumptions of normality and homogeneity of variance were verified. ANOVA was then conducted to evaluate treatment effects, and where significant differences among treatment means were detected, means were compared using the Least Significant Difference (LSD) test at the 5% and 1% probability levels.

### Cost–benefit analysis

A cost–benefit analysis was conducted employing prevailing market prices to assess the relative economic returns of the applied treatments. To account for potential discrepancies between research conditions and farmer realities, the marketable cabbage yield was conservatively adjusted downward by 10% before economic evaluation^31^. Farm service prices were sourced from the markets of Debre Markos and Rebu Gebeya in northwestern Ethiopia. For the purpose of calculation and subsequent analysis, the average of these values was utilized. The following economic indicators were employed in the evaluation:

**Gross benefit (GFB)**: This is the sum of the adjusted yield (t ha^− 1^) and the selling price. It was determined by multiplying the yield in tons per acre by the market price.

$$~GFB\left( {Gross~fieldbenefit/ha} \right)=Fram~gate~price/quintal*average~cabbage~yield)/ha)$$

**Net benefit (NB)**: NB was estimated by subtracting the total cost of production from the gross benefit.

$$NB=GFB - Total~cost$$

**Marginal analysis**: This metric compares net benefits to total variable costs, and the total variable cost for each treatment was calculated and compared to the net benefit.

**Dominance analysis**: Treatments were organized in ascending order based on their variable costs. The associated net benefits for each treatment are also presented. A treatment exhibiting a higher cost but yielding a lower net benefit compared to any preceding treatment is classified as economically dominated.

**Marginal Rate of Returns (MRR)**: The percentage change in benefit relative to the change in total variable cost when transitioning from a lower-cost to a higher-cost treatment was calculated to assess profitability. All treatments were ranked in descending order of profitability. This calculation involved multiplying the ratio of net benefit to total variable cost by 100.

$${\mathrm{MRR}}={\mathrm{\boldsymbol{\Delta}NB}}/{\mathrm{\boldsymbol{\Delta}}}TVC * 100$$

**Table 3:** Mean square values for the phenology, growth, yield, and quality components as influenced by apical bud manipulation and farmyard manure.

Locations	Factors	d.f	Parameters										
			PH	Hw	Ctn	MY	UY	TY	HI	HM	S	M	L
L_1_+L_2_	B	2	58.8*	1,061,770*	0.6*	243.5*	1.05*	224.35*	73.6*	230.9*	1.05*	142.8*	22.5*
	FYM	3	263.2*	3,883,817*	1.75*	693.95*	32.2*	464.55*	345.2*	440.5*	32.3*	362.9*	65.7*
	B*FYm	6	44.7*	1,286,077*	0.6*	179.15*	0.85*	179.45*	72.1*	222*	0.85*	113.7*	15.4*
	Rep	2	8.02	1,088,804	0.105	72.35	1.4	83.9	130.5	85.15	1.35	47	10.15
	Error	22	7.7	128441.3	0.035	21.4	1.22	22.6	15.9	17.15	1.25	11.5	3.05

ns = no significant difference; * = significant difference.

PH= Plant height, Hw= head weight, Ctn= Compactness index, MY= Marketable yield, UY=Unmarketable yield, TY= Total yield, HI=Head initiation, HM= Head maturity, S= small, M=medium, and L= large sized heads.

## Results

This study investigated the combined effects of apical bud manipulation and FYM application on the growth and yield attributes of cabbage. The main and interactive effects of these factors on growth parameters and yield components are summarized in Tables 3 and 4.

### The effect of bud manipulation and FYM rate on the phenology of cabbage crops

#### Days to 50% head initiation and 90% maturity

The interaction between bud number and FYM application rate exerted a statistically significant effect (*p* < 0.05) on cabbage head initiation and maturity, as indicated by the analysis of variance (Table 3). Across both experimental sites, the combined treatment of two buds with 5 tons of FYM per hectare resulted in the earliest head initiation (56 days) and maturity (78.5 days). In contrast, the latest head initiation (83 days) and maturity (119.3 days) were observed in plants with three buds and no FYM application (Table 4).

These findings demonstrate that higher nutrient inputs delayed the onset of reproductive growth stages, whereas treatments with low or no nutrient application accelerated these phases. This outcome aligns with previous reports indicating that nitrogen and FYM amendments significantly shorten the duration to head initiation, with treated plants initiating heads around 57.45 days compared to controls.

Furthermore, days to head initiation exhibited an inverse relationship with nutrient rates, as evidenced by ANOVA results.

### The effect of bud manipulation and FYM rate on cabbage crop growth

#### Plant height (cm)

The combined effect of bud number and FYM application rate significantly influenced cabbage plant height (*p* < 0.05), as presented in Table 3. The greatest mean plant height (30.9 cm) was observed in the treatment integrating two buds with 5 tons of FYM per hectare. Conversely, the shortest plants (7.4 cm) occurred in the treatment with three buds and no FYM application, a result statistically comparable to several other treatments (Table 4).

Overall, the type of organic fertilizer exerted less influence on vegetative growth parameters such as plant height and leaf number compared to its pronounced effects on yield-related traits, including fresh and dry biomass, as well as quality attributes like head diameter and grading. As anticipated, the rate of organic fertilizer application significantly influenced nearly all evaluated parameters.

Notably, plant height tended to decline when the organic fertilizer rate was increased beyond 3 tons or decreased below 3 tons across both locations (Table 4).

### The effect of bud manipulation and FYM rates on cabbage crop yield

#### Head weight (g)

A statistically significant interaction effect (*p* < 0.05) between bud number and FYM application rate on cabbage head weight (g) was detected (Table 3). The highest mean head weight (3355 g) was achieved by the combined treatment of two buds and 5 tons of FYM t ha^− 1^, whereas the lowest head weight (217.3 g) was recorded from plants with three buds and no FYM application across both locations (Table 4). Compared to other treatments, the integration of the optimal FYM rate with two buds resulted in substantially greater head weights, markedly exceeding those of both the unfertilized control and the excessively fertilized treatments. Head weight demonstrated a significant positive response to manure application when combined with the optimal bud number.

#### Marketable yield (t ha^− 1^)

The interaction between bud number and FYM application rates significantly affected marketable cabbage yield at both study locations (*p* < 0.05) as detailed in Table 3. The highest marketable yield (41.8 t ha^− 1^) was recorded from the combined treatment of two buds with 5 tons of FYM t ha^− 1^, whereas the lowest yield (2.1 t ha^− 1^) was observed in plants with three buds and no FYM application at both sites (Table 4).

#### Unmarketable yield (t ha^− 1^)

The interaction between bud number and FYM application rate exhibited a statistically significant effect (*p* < 0.05) on the unmarketable yield of cabbage (Table 3). The highest unmarketable yield (6.3 t ha^− 1^) was observed in the treatment combining three buds with no FYM application, whereas the lowest unmarketable yield (1.3 t ha^− 1^) was recorded from the treatment with two buds and 7.5 tons of FYM across both locations (Table 4).

#### Total yield (t ha^− 1^)

The combined effect of bud number and FYM application rate on total cabbage yield was statistically significant (*p* < 0.05) as presented in Table 3. The highest total yield (43.1 t ha^− 1^) was achieved in both districts under the treatment integrating two buds with 5 tons of FYM t ha^− 1^. In contrast, the lowest total yield (7.9 t ha^− 1^) was recorded from the treatment with two buds and no FYM application, which was statistically comparable to the yield from three buds without FYM application (Table 4).

FYM application significantly enhanced total yield, compactness index, and other agronomic parameters relative to the unfertilized control (Table 4). Overall, the superior yield achieved with 5 t ha^− 1^ FYM combined with two-bud management can be attributed to the complementary effects of balanced nutrient supply and controlled bud load. At this application rate, FYM provides adequate nutrients to sustain vigorous growth without encouraging excessive vegetative development. Limiting the plant to two buds allows these nutrients and assimilates to be directed toward a smaller number of developing heads, enhancing their size and overall productivity. This interaction between optimal nutrient availability and efficient assimilate partitioning likely explains the markedly higher total yields observed under this treatment.

### The effect of bud manipulation and FYM rate on cabbage crop quality

#### Compactness index

The interaction between cabbage bud number and FYM application rate significantly influenced the compactness index (*p* < 0.05) as indicated in Table 3. The highest compactness index (2.7%) was achieved with the combined treatment of two buds and 5 t of FYM ha^− 1^ at both locations, whereas the lowest index (0.5%) was observed in the treatments involving two and three buds without FYM application (Table 4).

#### Cabbage head size distribution by weight (t ha^− 1^)

##### Small-sized heads

Both the main and interaction effects of bud number and FYM application rates exhibited statistically significant differences (*p* < 0.05) in the production of small-sized cabbage heads, as presented in Table 3. The lowest quantity of small heads (1.3 t ha^− 1^) was obtained from the combination of two buds and 7.5 t of FYM ha^− 1^. Conversely, the highest production of small heads (6.3 t ha^− 1^) was recorded under treatments involving two and three buds with no FYM application across both experimental locations (Table 4).

##### Medium-sized heads

A statistically significant difference (*p* < 0.05) was observed in the yield of medium-sized cabbage heads due to both the main and interactive effects of bud number and FYM application rates, as illustrated in Table 3. The highest yield of medium-sized heads (31.8 t ha^− 1^) was achieved through the integration of two buds with the application of 5 t of FYM ha⁻¹ across both experimental locations. In contrast, the lowest yield (2.1 t ha^− 1^) was recorded from the three-bud treatment with no FYM application (Table 4).

##### Large heads

The main and interaction effects of bud number and FYM application rates on the production of large cabbage heads demonstrated a statistically significant difference (*p* < 0.05) across both experimental sites (Table 3). The maximum yield of large-sized heads (14.8 t ha^− 1^) was obtained from the integrated treatment of two buds combined with 5 t of FYM ha^− 1^. In contrast, negligible yields were recorded under treatments involving two or three buds without any FYM application (Table 4).

**Table 4:** Growth, yield, and quality components of head cabbage as influenced by the interaction effects of bud manipulation and FYM.

Location	Bud	FYM	PH	Hw	Ctn.	HI	HM	S	M	L	MY	UY	TY
L_1_+L_2_	1	0	9.6^de^	_374.2_^bcd^	0.8^cde^	67.6^abc^	_96.8_^ab^	4.5^ab^	2.8^cde^	1.3^cd^	3.5^cd^	4.5^ab^	8.0^bc^
		2.5	17.0^bc^	_1300.7_^b^	1.4^b^	61.3^abc^	_94.3_^ab^	1.5^bc^	10.8^bcd^	5.0^b^	16.1^b^	1.5^bc^	17.6^bc^
		5	19.3^b^	_1203.6_^bcd^	1.3^bc^	63.7^abc^	_96.5_^ab^	1.4^bc^	12.1^b^	5.1^b^	17.3^b^	1.6^bc^	19.0^bc^
		7.5	17.2^bc^	_1129.6_^bcd^	1.2 ^bc^	57.3^ab^	_93.8_^ab^	1.7^bc^	7.4^bcde^	3.2^bcd^	11.0^bcd^	1.7^bc^	12.8^bc^
	2	0	10.5^cde^	_289.3_^bcd^	0.5^de^	70 ^bc^	_101.5_^b^	5.1^a^	2.8^cde^	0.0^d^	2.8^cd^	5.1^a^	7.9^bc^
		2.5	16.4^bc^	_1231.3_^bcd^	1.4^b^	64.7^abc^	_96.7_^ab^	1.4^bc^	12.6^bc^	5.8^b^	18.6^b^	1.4^bc^	20.0^b^
		5	30.9^a^	3355^a^	2.7^a^	56^a^	78.5^a^	1.5^bc^	31.8^a^	14.8^a^	41.8^a^	1.3^bc^	43.1^a^
		7.5	16.8^bcd^	_968.3_^bcd^	1.2^bc^	63^abc^	_97.1_^ab^	1.3^bc^	10^bcde^	5.5^b^	15.1^bc^	1.3^bc^	16.5^bc^
	3	0	7.4^e^	_217.3_^cd^	0.5^de^	83^c^	_119.3_^c^	6.3^a^	2.1^de^	0.00^d^	2.1^cd^	6.3^a^	8.45^bc^
		2.5	17.6^b^	_1081.6_^bcd^	1.2^bc^	62.2^abc^	_95.9_^ab^	1.6^bc^	10.6^bcd^	2.9^bcd^	14.1^bc^	1.6^bc^	15.7^bc^
		5	16.4^bc^	_1120.3_^bcd^	1.1^bcd^	60.4^abc^	_94.0_^ab^	1.7^bc^	9.8^bcde^	3.7^bc^	12.8^bcd^	1.7^bc^	14.6^bc^
		7.5	16.1^bcd^	_1199.3_^bcd^	1.3^b^	63.3^abc^	_94.8_^ab^	1.7^bc^	12.5^bcd^	6.2^b^	17.3^b^	1.7^bc^	19.0^b^
LSD (0.05)			6.2	1042.	0.4	11.4	10.8	2.5	8.5	3.5	11.5	2.4	11.9
CV (%)			16.7	32.2	16.2	6.1	4.6	39.4	32.5	34.8	32.2	38.1	28.2

LSD = least significant difference, CV (%) = coefficient of variation.

Table 5 Standard deviation values (±) of cabbage crop growth, yield, and quality components as influenced by bud manipulation and FYM.

**Table 5:** Standard deviation and error values (±) of cabbage crop growth, yield, and quality components as influenced by bud manipulation and FYM.

Bud	FYM (t ha^–1^)	PH (cm)	Hw	Ctn.	HI	HM	S	M	L	MY	UY	TY
1	0	2.39 ± 1.38	51.85 ± 29.95	0.35 ± 0.20	2.41 ± 1.39	27.97 ± 16.14	0.86 ± 0.50	1.21 ± 0.70	1.21 ± 0.70	2.22 ± 1.28	0.86 ± 0.50	2.34 ± 1.35
	2.5	3.21 ± 1.85	550.84 ± 318.05	0.18 ± 0.10	5.14 ± 2.97	32.39 ± 18.70	0.43 ± 0.25	3.44 ± 1.99	2.44 ± 1.41	4.74 ± 2.74	0.35 ± 0.20	4.60 ± 2.66
	5	2.97 ± 1.71	494.48 ± 285.49	0.24 ± 0.14	2.47 ± 1.43	32.01 ± 18.49	0.38 ± 0.22	3.39 ± 1.96	1.55 ± 0.90	4.32 ± 2.50	0.40 ± 0.23	4.64 ± 2.68
	7.5	2.19 ± 1.26	622.36 ± 359.15	0.14 ± 0.08	5.26 ± 3.04	35.83 ± 20.68	0.93 ± 0.54	4.64 ± 2.68	3.21 ± 1.85	7.11 ± 4.10	0.93 ± 0.54	7.28 ± 4.20
2	0	2.80 ± 1.62	38.87 ± 22.45	0.19 ± 0.11	1.50 ± 0.87	30.46 ± 17.58	1.27 ± 0.73	0.66 ± 0.38	0.00 ± 0.00	0.66 ± 0.38	1.27 ± 0.73	1.47 ± 0.85
	2.5	3.11 ± 1.80	397.14 ± 229.24	0.19 ± 0.11	3.89 ± 2.25	31.14 ± 17.98	0.50 ± 0.29	3.77 ± 2.18	1.38 ± 0.80	4.92 ± 2.84	0.50 ± 0.29	5.31 ± 3.07
	5	5.43 ± 3.13	230.10 ± 132.88	0.35 ± 0.20	7.17 ± 4.14	21.61 ± 12.47	0.77 ± 0.44	4.63 ± 2.67	6.08 ± 3.51	4.79 ± 2.77	0.29 ± 0.17	4.93 ± 2.85
	7.5	1.48 ± 0.85	618.98 ± 357.20	0.25 ± 0.14	4.15 ± 2.40	33.56 ± 19.38	0.61 ± 0.35	4.33 ± 2.50	1.87 ± 1.08	5.41 ± 3.12	0.62 ± 0.36	5.37 ± 3.10
3	0	1.37 ± 0.79	69.01 ± 39.86	0.37 ± 0.21	1.39 ± 0.80	35.82 ± 20.69	2.92 ± 1.69	1.02 ± 0.59	0.00 ± 0.00	1.02 ± 0.59	2.92 ± 1.69	3.82 ± 2.21
	2.5	3.90 ± 2.25	370.41 ± 213.69	0.17 ± 0.10	6.08 ± 3.51	33.48 ± 19.34	0.49 ± 0.28	3.52 ± 2.03	1.36 ± 0.79	3.26 ± 1.88	0.49 ± 0.28	3.32 ± 1.92
	5	4.12 ± 2.38	550.73 ± 318.15	0.15 ± 0.09	6.68 ± 3.86	33.18 ± 19.15	0.45 ± 0.26	5.10 ± 2.94	2.38 ± 1.37	7.34 ± 4.24	0.46 ± 0.27	7.30 ± 4.22
	7.5	4.48 ± 2.59	489.75 ± 282.88	0.26 ± 0.15	4.83 ± 2.79	31.02 ± 17.91	0.32 ± 0.18	5.38 ± 3.11	2.15 ± 1.24	6.17 ± 3.56	0.32 ± 0.18	5.90 ± 3.41
LSD (0.05)	-	6.2	1042	0.4	11.4	10.8	2.5	8.5	3.5	11.5	2.4	11.9
CV (%)	-	16.7	32.2	16.2	6.1	4.6	39.4	32.5	34.8	32.2	38.1	28.2

The combined application of Bud and FYM improved plant height, head traits, and overall yield compared with the untreated control (Table 5). Overall, higher FYM rates under Bud levels 2 and 3 produced consistently greater marketable yields.

### Cost–benefit analysis of cabbage production as influenced by bud manipulation and FYM rate

For the economic analysis, the mean yield from each treatment was utilized rather than the cumulative data from all replications^31,32^. A net benefit of 1917.59 USD was achieved through the combined application of two cabbage buds and 5 t ha^− 1^ of FYM, followed by a net benefit of 850.77 USD from the treatment involving two buds and 2.5 t ha^− 1^ of FYM. In contrast, the lowest net return, amounting to 98.70 USD, was recorded from the treatment with three buds and no farmyard manure application (Table 6). Both total costs and net benefits showed a progressive increase across treatments up to a certain threshold.

The economic analysis demonstrated that integrating bud number regulation with FYM application substantially improved the profitability of cabbage production. The combination of two buds with 5 t ha^− 1^ FYM generated the highest net benefit, indicating that this treatment achieved an optimal balance between input cost and yield gain. In contrast, lower returns from treatments without FYM or with excessive bud numbers highlight the economic inefficiency of suboptimal nutrient supply and uncontrolled bud load. These findings suggest that moderate FYM application paired with strategic bud reduction not only enhances biological productivity but also maximizes financial returns, making it a practical and economically viable option for smallholder cabbage producers.

**Table 6:** Economic analysis of cabbage as influenced by bud manipulation and FYM.

L	Bud + FYM	IC Per ha^− 1^ FYM ( USD)	AC ( USD )	LC for mgt. ( USD )	TVC ( USD )	MY (t ha^− 1^)	ADY (t ha^− 1^)	GI	NB	Rank
L_1_ & L_2_	1:0	0.00	0.00	0.00	0.00	3.5	3.15	163.63	163.63	10
	1:2.5	16.23	1.62	0.00	17.85	16.1	14.49	752.72	734.87	5
	1:5	32.46	3.24	0.00	35.71	17.3	15.57	808.83	773.11	3
	1:7.5	48.70	4.87	0.00	53.57	11.0	9.9	514.28	460.71	9
	2:0	0.00	0.00	-0.97	-0.97	2.8	2.52	130.90	129.93	11
	2:2.5	16.23	1.62	-0.97	18.83	18.6	16.74	869.61	850.77	2
	2:5	32.46	3.24	-0.97	36.68	41.8	37.62	1954.28	1917.59	1
	2:7.5	48.70	4.87	-0.97	54.54	15.1	13.59	705.97	651.42	6
	3:0	0.00	0.00	1.29	1.29	2.1	1.89	98.18	96.88	12
	3:2.5	16.23	1.62	1.29	19.15	14.1	12.69	659.22	640.06	7
	3:5	32.46	3.24	1.29	37.01	12.8	11.52	598.44	561.42	8
	3:7.5	48.70	4.87	1.29	54.87	17.3	15.57	808.83	753.96	4

**Table 7:** Dominance analysis for cabbage yield as affected by bud manipulation and FYM in northwestern Ethiopia.

Bud + FYM	TVC (USD ha^-1^)	NB. (USD ha^-1^)	B: C ratio
1:0	0.00	163.63	-
2:0	-0.97	129.93D	133.4
3:0	1.29	96.88D	74.6
1:2.5	1.78	734.87	41.15
2:2.5	18.83	850.77	45.17
3:2.5	19.15	640.06D	33.41
1:5	35.71	773.11D	21.64
2:5	36.68	1917.59	52.26
3:5	37.01	561.42D	15.16
1:7.5	53.57	460.71D	8.6
2:7.5	54.54	651.42D	11.94
3:7.5	54.87	753.96D	13.74

B: C= Benefit-cost ratio.

The comparatively high B: C ratios observed in certain treatments (Table 7) result from the substantially greater net benefits obtained relative to the corresponding total variable costs. These values are reflecting the strong economic advantage of treatments that produced higher yields without proportional increases in production costs.

Marginal rate of return (MRR) (Table 8) analysis also showed clear economic gains from Bud*FYM combinations. The 2:5 treatment provided the highest return.

**Table 8:** Marginal rate of return (MRR) of cabbage yield as affected by bud manipulation and FYM in northwestern Ethiopia.

Treatment combinations	TVC (USD ha^− 1^)	MC (USD ha^− 1^)	NB (USD ha^− 1^)	MB (USD ha^− 1^)	MRR (%)
1:0	0.00		163.63		
1:2.5	17.85	17.85	734.87	571.23	20.76
2:2.5	18.83	-0.97	850.77	115.90	77.27
2:5	36.68	17.85	1917.59	1066.81	38.79

MC= marginal cost, MB= marginal benefit, MRR= marginal rate of return.

## Discussion

### The influence of bud manipulation and FYM rate on the phenology of cabbage crops

#### Days to 50% head initiation and 90% maturity

The time required for 50% of cabbage plants to initiate head formation shortened consistently as the levels of combined nutrients increased. These results show that better nutrient availability accelerates head formation, confirming that adequate fertility promotes timely cabbage development^33^. Similar findings in previous studies have shown that enhanced nutrient supply improves vegetative growth and triggers earlier reproductive development in cabbage^5^. Conversely, plants that did not receive any fertilizer took a significantly longer period, averaging 77.0 days, to begin head formation^34,35^. This delay is likely due to limited availability of essential nutrients required for rapid vegetative growth. In contrast, plants receiving adequate fertilization had faster metabolic activity, enabling earlier initiation of reproductive development^36^. Likewise, cabbage plants supplied with either organic or inorganic fertilizers exhibited a reduced duration for head initiation^37^.

Typically, cabbages treated with the highest rates of FYM reached maturity sooner than those that received little or no FYM^38^. Furthermore, these findings align with previous reports indicating that the combined application of organic and inorganic nutrients can lead to a delay in plant maturity^39,40^.

Compared with FYM alone, higher nutrient availability hastened physiological maturity, with well-nourished plants maturing earlier than the untreated controls^41^. Organic manure also extended vegetable longevity by improving nutrient uptake and strengthening water-conducting tissues^42^. Fertilizer application influenced the maturity of cabbage heads, with treated plants reaching maturity earlier than those that received no fertilization.

### The effect of bud manipulation and FYM rate on cabbage crop growth

#### Plant height (cm)

The increased plant height observed under the two-bud treatment with FYM is likely due to improved soil conditions, such as greater nutrient availability, better water-holding capacity, and an enhanced soil structure^43^. FYM has the potential to promote plant growth, likely due to the presence of growth-regulating substances such as auxins, cytokinins, and gibberellins in the fertilizer^44^. Exclusive reliance on inorganic fertilizers can degrade soil health, whereas incorporating FYM and other organic sources is more effective for improving cabbage height^45^. Organic fertilizers can serve as an alternative to mineral fertilizers, enhancing soil structure and supporting microbial biomass. In particular, FYM promotes soil microbial activity, which increases nutrient availability for bud development and ultimately contributes to higher yields^46^. The data indicate that plant height increases as the FYM rate is elevated under the two-bud treatment. Consistent with these findings, cabbage height was observed to be greater with higher FYM application rates^47^. Furthermore, higher applications of nitrogen combined with FYM contribute to an increase in plant height^48^. Similarly, other studies have shown that the application of bio-manure enhances plant height by providing a steady nutrient supply^49,50^. Among the nutrients, nitrogen and phosphorus in the enriched residue play a pivotal role in promoting vigorous vegetative growth^51^. This improved nutrient availability supports more robust stem and leaf development, ultimately contributing to taller plants^52^. Overall, the increase in plant height with FYM application is likely due to improved soil structure and aggregation, which enhance nutrient availability^53^.

### The effect of bud manipulation and FYM rate on cabbage crop yield

#### Head weight (g)

Cabbage head weight exhibited variability influenced by the combined effect of farmyard manure application rates and the number of buds^54^. Head weight is a key parameter for assessing yield performance. In line with this, applying higher rates of farmyard manure alongside an optimal number of buds led to an increase in cabbage head weight^55^. Moreover, the use of organic matter markedly enhanced growth parameters, promoting greater synthesis of plant metabolites and ultimately boosting yield^56^.

As the application rate of farmyard manure rises to an optimal level, head mass correspondingly increases. Additionally, organic manure stimulates diverse soil microorganisms, which release phytohormones that can enhance plant growth and nutrient uptake^57^.

Thus, nitrogen supports microbial activity, helping to explain why combining FYM with inorganic fertilizer improved both growth and yield^58^. Consequently, nitrogen supports microbial proliferation, helping explain why combining FYM with inorganic fertilizer improved overall growth and yield^59,60^. The additional nutrients from farmyard manure provide a sustained release of nitrogen, creating a favorable environment for microbial activity and plant development^61^. Moreover, integrating organic and inorganic fertilizers can improve soil fertility and structure, further promoting efficient nutrient uptake and higher crop productivity^62^.

#### Marketable yield (t ha^− 1^)

Marketable yield serves as a key factor guiding farmers’ choices in cabbage cultivation. Yield improvements at higher organic fertilizer levels likely result from balanced nutrient supply that enhances cell expansion, boosts vegetative growth, improves photosynthesis, and supports efficient assimilate translocation to developing heads^63,64^. In this study, applying 5 t ha^− 1^ FYM together with two-bud management greatly increased marketable yield by supplying key macronutrients and essential micronutrients. The two-bud approach likely reduced intra-plant competition for light, nutrients, water, and space, as compared with the three-bud setup, thereby optimizing resource use efficiency. All tested treatment combinations produced higher yields than the unfertilized control, highlighting the benefits of organic amendments.

Most importantly, the superior performance of the 2-bud treatment can be attributed to optimized source–sink balance, where limiting the number of active buds reduces competition for assimilates, allowing more resources to be allocated to the remaining buds and promoting vigorous growth and higher yield. FYM further enhances growth, particularly under acidic soil conditions, by improving soil structure, increasing nutrient availability, and buffering soil pH, which facilitates better root development and nutrient uptake. The combined effect of strategic bud reduction and FYM application thus maximizes both vegetative growth and yield potential by ensuring efficient resource allocation and improved soil fertility. Conversely, the combination of excessive FYM with an increased number of buds did not result in proportional yield gains, potentially due to nutrient imbalances or intensified competition among plant parts^65^. These findings agree with earlier reports showing that organic fertilizers markedly improve vegetable yields, highlighting the need to optimize both nutrient supply and plant management for maximum productivity^66^. FYM also provides organic carbon and a broad range of essential nutrients including N, P, K, Ca, Mg, and Fe that collectively support sustained and healthy crop development^67^.

#### Unmarketable yield (t ha^− 1^)

In general, treatments that did not receive nutrient supplementation consistently produced the lowest marketable yields^68^. Even the minimum yield per hectare could be attained from any of the control treatments in cabbage production^69^. Crops in nutrient-deficient conditions exhibited stunted growth and smaller biomass, resulting in fewer harvestable products^70^. Moreover, nutrient deficiencies led to smaller produce, reduced quality, delayed maturity, and ultimately lower total yield^71,72^.

#### Total yield (t ha^− 1^)

The addition of FYM substantially enhanced the soil’s structural, chemical, and biological characteristics, creating a more favorable environment for optimal crop growth^73^. By enriching the soil with organic matter, FYM improved water retention and aeration, which supported healthy root development^61^. Its gradual release of nutrients increased soil fertility, ensuring a steady supply of essential elements for the crops. In addition, FYM stimulated beneficial microbial activity, strengthening the soil ecosystem and enhancing plant nutrient uptake and resilience against stress^74^. Collectively, these improvements in soil health contributed to significantly better crop performance^75^. Increasing the organic carbon content in soil markedly enhances the availability of essential nutrients, fostering a more fertile and resilient soil environment^76^. In cabbage production, bud regulation is emerging as a useful management practice, while determining the optimal FYM rate remains a key focus for improving field performance^77^. As such, FYM remains indispensable in soil management strategies, effectively tackling major limitations commonly encountered in tropical agricultural systems^78^. Increased organic matter stimulates microbial activity within the soil, which in turn accelerates nutrient cycling and fosters robust plant growth^79^. Regular FYM application supports long-term crop sustainability by maintaining soil health and improving resilience to environmental stresses^80^. Organic manure reduces dependence on synthetic fertilizers while supplying essential nutrients, and it also enhances soil structure, fertility, and water-holding capacity, and creating a healthier and more productive soil environment^81^.

### The effect of bud manipulation and FYM rate on cabbage crop quality

#### Compactness index

Head compactness is important in cabbage production because it enables higher yield efficiency by providing more harvestable volume from smaller heads^82^. The incorporation of FYM markedly improved several key morphological traits, including plant height, stem thickness, head diameter, head density, and the roundness of the head^83^. These findings state the strong connection between improved plant morphology and yield, indicating the role of FYM in supporting vigorous crop growth.

Furthermore, improved head compactness contributes to better marketable quality, making the crop more appealing to consumers^84^. Improved structural growth from FYM application increases plant resilience to environmental stress and mechanical damage during harvest^85^. Consistent use of organic amendments like FYM can therefore lead to sustainable productivity gains while maintaining the structural and nutritional integrity of cabbage heads^86^. Application of FYM resulted in notable increases in both the diameter and density of cabbage heads, reflecting a pronounced improvement in overall head development^87^. Cabbage plants receiving FYM showed clear increases in height, head length, head diameter, and compactness, reflecting substantial improvements in morphological development^88^.

### **Cabbage head size distribution by weight (**t ha^− 1^**)**

#### Small-sized heads

Smaller cabbage heads are generally favored in fresh produce markets because their compact size aligns with consumer preferences for convenient handling and portioning^89^. Desirable head size can be achieved through appropriate cultivar selection and optimized agronomic practices, including moderate fertilization that matches the crop’s nutrient needs^90^. This balanced nutrient management approach ensures that growth is directed toward quality enhancement rather than excessive vegetative expansion^91^.

Furthermore, producing market-preferred head sizes contributes to reduced post-harvest losses and improved storage stability^92^. Such practices also promote uniformity in crop appearance, enhancing both market value and consumer acceptance. By fine-tuning fertilizer application in line with cultivar characteristics, growers can achieve an ideal balance between yield efficiency and commercial desirability^93,94^.

#### Medium-sized heads

Medium-sized cabbage heads constitute a vital portion of the marketable yield and hold significant economic value for producers^95^. The improved head size distribution with FYM and optimized agronomic practices likely reflects enhanced physiological processes. These improvements likely result from stronger root growth and more efficient assimilate translocation, supported by bioactive compounds released as organic matter decomposes^96^.

In addition, the improved nutrient availability from FYM promotes sustained vegetative growth and supports uniform head formation^97^. Enhanced soil conditions resulting from organic amendments also create a favorable microenvironment that reinforces plant vigor and yield consistency^98^. The specific type of organic fertilizer used appeared to have a limited impact on cabbage head development, whereas the application rate showed a pronounced positive effect on key quality traits^99^. Notably, higher rates of organic fertilizer, irrespective of the source, consistently promoted increases in both head size and diameter. These results suggest that total nutrient input, rather than the specific type of organic amendment, plays the dominant role in improving cabbage head morphology and marketable quality under the given conditions^100^.

#### Large heads

The highest yield of large-sized cabbage heads was achieved when two buds were managed in combination with the application of 5 t of FYM. Conversely, treatments involving two or three buds without FYM supplementation resulted in minimal head yields. These results show that nutrient availability is critical, with appropriate fertilization strongly influencing cabbage morphology, especially head formation and size distribution^39,101^. Regardless of plant population density, the use of FYM consistently improved the distribution of cabbage head sizes^34^. The interaction of bud number and FYM rate significantly shaped head size categories (0.5–1.5 kg), indicating their combined role in improving head uniformity and marketability^89^. Growing cabbage at higher plant densities helps to produce more uniform head sizes and lighter outer leaves, traits that are generally preferred by consumers^87^. The effects of plant spacing and fertilizer type on the growth and productivity of diverse vegetable crops have been extensively investigated. Research also shows that higher plant densities often produce smaller heads, highlighting the need for careful spacing and adequate nutrient supply to achieve optimal head size^89,102^.

## Conclusion

Integrating apical bud retention (two buds per plant) with moderate farmyard manure application (5 t ha^− 1^) proved to be a novel and effective strategy for cabbage cultivation in northwestern Ethiopia. This combination significantly enhanced yield (up to 43.1 t ha^− 1^), improved head-size uniformity, and delivered the highest net economic return (191759 USD ha^− 1^). The study demonstrates that harmonizing simple cultural practices with organic nutrient management offers a sustainable, and low-cost alternative to input-intensive systems, with strong potential for adoption in mid-altitude and similar agro-ecological regions.

## Supplementary Information

Below is the link to the electronic supplementary material.

Supplementary Material 1

## Data Availability

The dataset that supports the findings of this research is shared as a supplementary file.
